# Functionalization of Polyvinylpyrrolidone Films by Grafting Maleic Acid from PVP Gels for Loading Studies of Naringin and Silver Nanoparticles as Potential Wound Dressings

**DOI:** 10.3390/gels11020147

**Published:** 2025-02-19

**Authors:** Miguel S. Pérez-Garibay, Gabriel Ángel Lara-Rodríguez, Emilio Bucio

**Affiliations:** 1 Departamento de Química de Radiaciones y Radioquímica, Instituto de Ciencias Nucleares, Universidad Nacional Autónoma de México, Circuito Exterior, Ciudad Universitaria, Mexico City 04510, Mexico; 2 Laboratorio Materiales Metálicos Avanzados, Instituto de Investigaciones en Materiales, Universidad Nacional Autónoma de México, Circuito Exterior, Ciudad Universitaria, Mexico City 04510, Mexico; laragab@unam.mx

**Keywords:** polymer response to pH, γ-ray pre-irradiation method, AgNPs antimicrobial activity, maleic acid, PVP, naringin

## Abstract

Wound healing is a complex process involving stages such as hemostasis, inflammation, proliferation, and remodeling. In this context, polymers are useful materials for wound treatment. This research used the Casting method to prepare films from 2% polyvinylpyrrolidone (PVP) gels. Subsequently, PVP films were grafted with maleic acid (MA) (PVP-g-PAM) to load naringin (NA) and silver nanoparticles (AgNPs) in order to obtain a material with pH responsiveness and antibacterial properties. The modified PVP-g-PAM films were prepared using gamma-ray irradiation through a pre-irradiation oxidative method at a dose rate of 13.7 kGy h^−1^, doses ranging from 10 to 25 kGy, and reaction times from 50 to 80 min in a bath of water, all samples at 50 °C, and a fixed monomer concentration of 15% (*w*/*v*) MA in THF. The conditions that yielded the highest percentage of grafting were 20 kGy and 60 min. NA was loaded at a fixed concentration of 5%. Data release showed that the films follow the Korsmeyer-Peppas kinetic model. Synthesis of AgNPs was performed by γ-ray irradiation–reduction (10 and 30 kGy), using PVP as a stabilizer. AgNPs showed in vitro effectiveness against *E. coli* and *S. aureus*. Films were characterized by FTIR-ATR, TGA, DSC, mechanical properties, swelling index, and contact angle. Further studies must be implemented; however, the results up now suggest that PVP-g-PAM loaded with NA and AgNPs can be useful as a potential wound dressing.

## 1. Introduction

Polymers have become essential for therapeutic applications due to their various properties, such as durability, malleability, biodegradability, biocompatibility, and ease of processing. As a result, they represent the largest and most versatile class of biomaterials [1]. This versatility is attributed to the relative ease with which polymers can be designed and prepared with a wide range of structures, as well as specific physical, chemical, surface, and biomimetic properties [2]. These capabilities enable the creation of “smart polymers” that can respond to various stimuli, including pH, temperature, redox conditions, enzymes, light, magnetic fields, and ultrasound [3].

Several methods exist for modifying the properties of polymers, such as blending, grafting, and curing [4]. Grafting is a particularly useful technique for tailoring special functional groups and producing polymers with enhanced properties, such as improved wettability, biocompatibility, and mechanical strength [5].

In this context, gamma ionizing radiation (γ-ray irradiation) is defined as photons or particles with energy capable of ionizing atoms and/or molecular segments of covalent compounds [6], allowing the obtaining of graft polymers. In addition, γ-ray irradiation offers certain advantages over other methods, such as not requiring the use of catalysts or additives to initiate the reaction, providing rapid and uniform creation of active radical sites, and different techniques can be used [7]. The number and length of grafted chains can be controlled by selecting the dose and intensity of irradiation; temperature is not a determining factor in the percentage of grafting; relative easy preparation (compared to conventional chemical methods); and applicable to most polymers because energy absorption is not selective [8]. As a result, γ-ray irradiation has gained great interest in the past few decades because it allows the functionalization of different polymers, resulting in useful materials for many applications [9].

Polyvinylpyrrolidone (PVP)-based hydrogels are one of the most common due to interesting properties such as biocompatibility, biodegradability, low toxicity [10], low immunogenicity, antigenicity [11], water solubility [12], excellent transparency [13], good adhesion, and high hygroscopicity [14]. These attributes have led to extensive research on PVP in fields such as pharmaceutical and biomedical applications in tissue regeneration [15,16], wound healing [13,17,18,19], and controlled drug delivery systems [20,21]. Nonetheless, numerous PVP-based materials lack antibacterial properties. To address this, silver nanoparticles (AgNPs) are an option. AgNPs have been studied in several areas, such as dental, pharmaceutical, film, and food packaging purposes [22]. AgNPs help to prevent infections by inhibiting the growth of microorganisms [23], and they present strong inhibitory or bactericidal effects for a broad spectrum of bacteria, fungi, and viruses [24]. Moreover, unlike conventional chemical antimicrobial agents, AgNPs do not trigger resistance [23,25].

Maleic acid (MA) is a multifunctional organic compound that has recently gained interest in synthesizing polymers [26]. MA has been studied in various materials, such as microparticles [26], hydrogels [27,28,29], and copolymers [30,31], due to its structure, which exhibits a response to pH changes [28,30,32,33,34].

Naringin (NA) is a flavanone glycoside in citrus fruits and grapes [35]. It has been reported to possess various properties, including antioxidant, anti-inflammatory, anti-apoptotic, anti-cancer, anti-allergic, and angiogenic effects [36,37,38,39,40]. Due to these properties, NA has been widely studied as a wound-healing agent in various forms, including ointments [41], dressings [42], and oral preparations [43].

Wound healing is a complex process that can be supported by biomaterials. Still, many of the current polymer-based dressings lack sufficient antibacterial effects or do not adapt well to environmental changes such as pH. To overcome these limitations, this research focuses on the creation of a new polyvinylpyrrolidone (PVP)-based material incorporating silver nanoparticles (AgNPs) for antimicrobial activity, maleic acid (MA) for pH response, and naringin (NA) for its angiogenesis effects and anti-inflammatory properties.

## 2. Results and Discussion

### 2.1. Grafting of PVP-g-PAM

**Figure 1:**
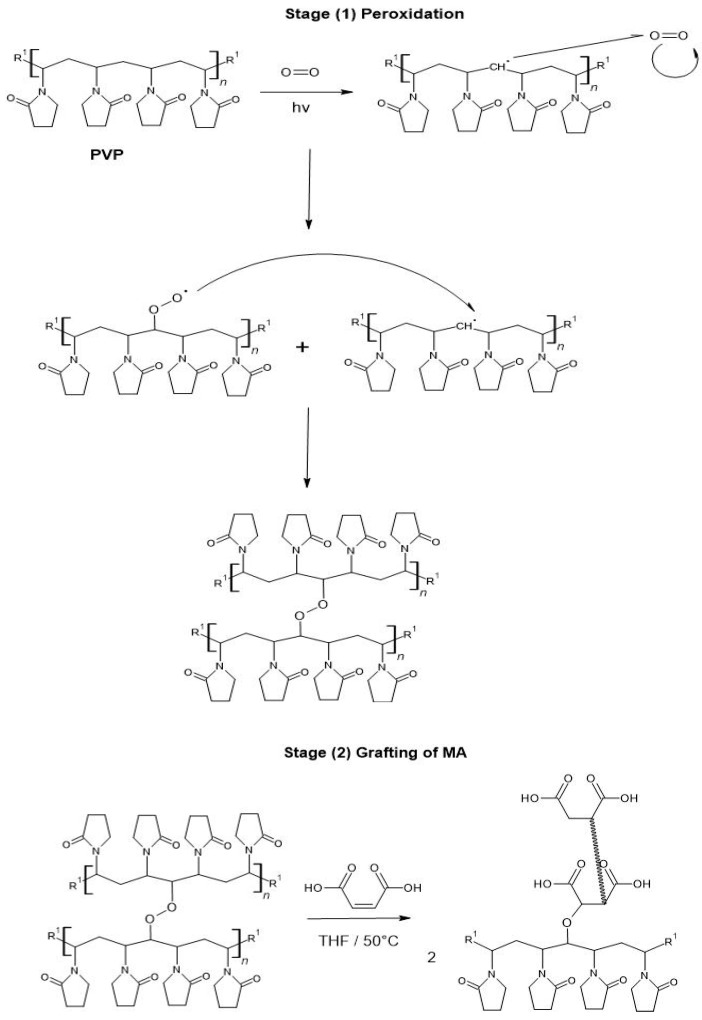
Proposed mechanism for the grafting of MA onto PVP films.

Table 1 presents the results of grafting AM onto PVP films, showing the percentages of AM grafting onto PVP films at varying irradiation doses. Table 2 presents the grafting percentages at different times in bath water. When varying the dose, the highest percentage of grafting (25%) occurs at 20 kGy, which is attributed to optimal peroxide formation (Figure 1). At doses of 25 kGy or higher, two factors contribute to a decrease in grafting: (1) degradation of the polymer matrix [44], and (2) excessive peroxide formation, leading to reactions between themselves reducing AM grafting.

**Figure 2:**
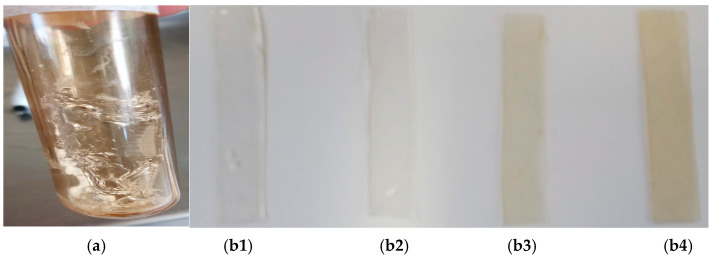
(**a**) PVP-g-PAM at 80 min in bath water. (**b1**) PVP film. (**b2**) PVP-g-PAM. (**b3**) PVP-g-AM-loaded NA. (**b4**) PVP-g-PAM-loaded NA + AgNPs.

When time varies, the grafting percentage increases to 31% at 60 and 70 min. This increase is due to the greater extent of reaction between the peroxides formed during irradiation and MA and the longer exposure time, allowing MA more time for in situ polymerization, as has been reported previously [45,46]. At 80 min, the film begins to dissolve in the medium (Figure 2a), causing not all of the film to be completely extracted, which results in a lower final weight record (compared to 50–70 min). In this research, we ascribe that the grafting of MA mainly occurs because we have a polymeric matrix with peroxides, which, due to their length, are likely to break by reacting with the monomer more easily. Figure 2(b1–b4) illustrates the color change of the PVP film after the AM grafting process, followed by the subsequent loading steps of NA and AgNPs.

### 2.2. Synthesis of AgNPs

**Figure 3:**
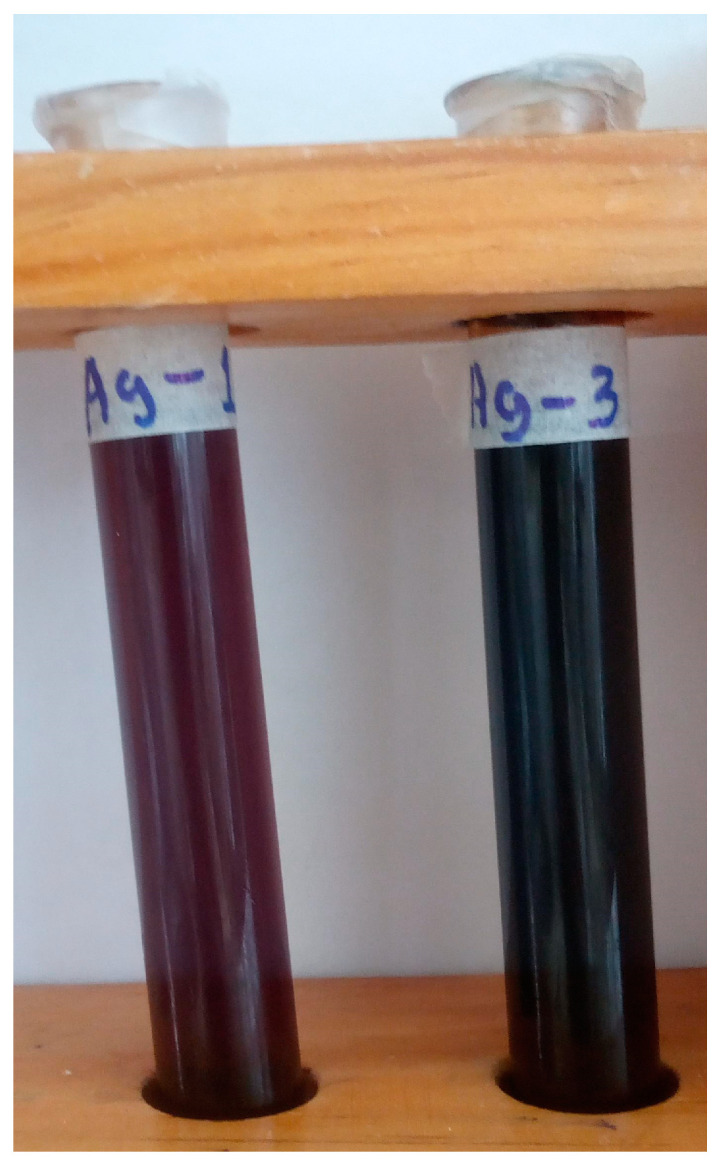
AgNPs obtained by γ-ray irradiation reduction of silver nitrate. Left 10 kGy. Right, 30 kGy.

**Figure 4:**
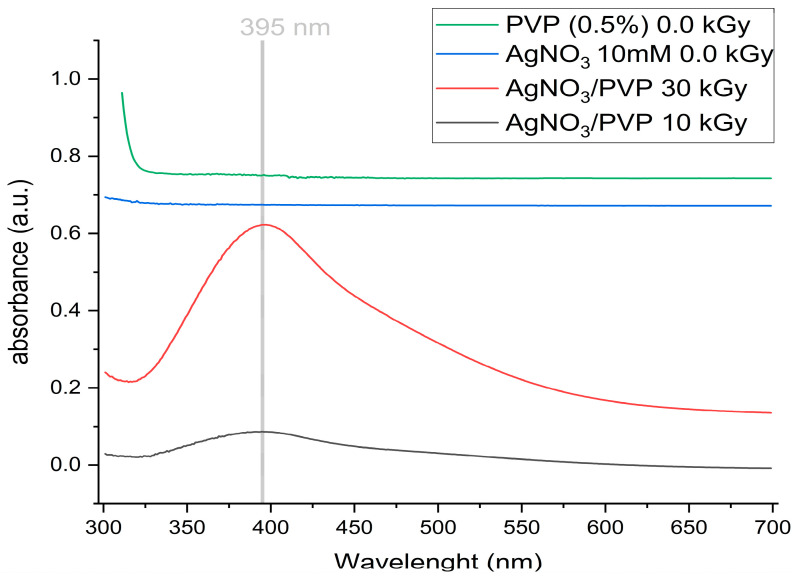
UV spectra of AgNPs/PVP obtained by γ-ray irradiation reduction of silver nitrate.

AgNPs were synthesized by γ-ray irradiation reduction of silver nitrate in a PVP solution. Before the irradiation process, each sample (10 and 30 kGy) was clear and transparent. After irradiation, the samples turned brown (Figure 3), indicating the formation of silver nanoparticles and silver oxide nanoparticles [22,47,48]. UV-Vis spectroscopy suggests the formation of AgNPs, showing a maximum absorption band at 395 nm (Figure 4), which matches the plasmon values reported in literature around 400 nm [48,49,50]. However, techniques such as atomic force microscopy (AFM), transmission electron microscopy (TEM), scanning electron microscopy (SEM), or/and dynamic light scattering (DLS) are necessary to confirm AgNPs. Both samples remained stable for 6 months. This stability is attributed to the presence of PVP, which enhances the nucleation of AgNPs and effectively stabilizes the dispersed particles [47,51,52].

### 2.3. Fourier Transform Infrared Spectroscopy with Attenuated Total Reflectance (FTIR-ATR)

**Figure 5:**
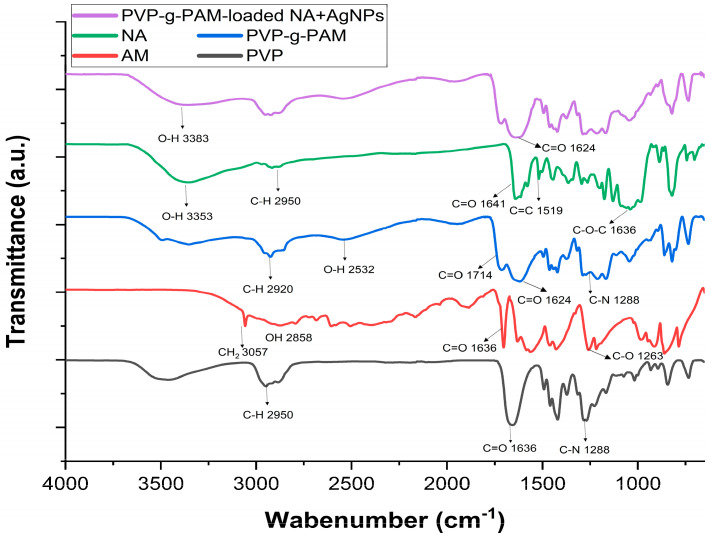
FTIR−ATR spectra of PVP, PVP-g-PAM (31.8% grafting of MA), PVP-g-PAM-loaded NA + AgNPs (31.8% grafting of MA), AM, and NA.

Figure 5 illustrates the spectra for different samples. The PVP film shows a band at ~2950 cm^−1^, assigned to the C–H stretching vibration, at ~1636 cm^−1^, ascribed to C=O stretching [50], and at ~1288 cm^−1^, attributed to the C–N stretching vibration [20]. MA presents bands at ~3057, ~2858, ~1704, and ~1263 cm^−1^, which correspond to the C–H stretching bond of CH_2_, O–H stretching, C=O stretching, and C−O stretching, respectively [26]. NA exhibits bands at ~3353, ~2916, ~1641, ~1519, and ~1040 cm^−1^, assigned to O–H stretching, C–H stretching bond of CH_3_, C=O stretching, C=C stretching bond of the aromatic ring, and C–O–C stretching, respectively [53,54]. The spectrum of PVP-g-PAM confirms the grafting of MA onto the PVP films, showing bands at ~2920, ~2532, ~1714, ~1624, and ~1288 cm^−1^, which are attributed to the C–H stretching vibration of PVP, O–H stretching of MA, C=O stretching of MA, C=O stretching of PVP, and C–N stretching vibration of PVP, respectively. Additionally, the band at ~3057 cm^−1^ from MA disappears due to the in situ polymerization of MA. Finally, the spectrum of PVP-g-PAM-loaded NA + AgNPs corroborates the presence of NA and silver nanoparticles. Bands between 3635 and 3033 cm^−1^, with a maximum of ~3383 cm^−1^, and bands between 1695 and 1520 cm^−1^, with a maximum of ~1637 cm^−1^, become broader (with respect to PVP-g-PAM) in both cases. The first case is attributed to the presence of NA, causing the bands of PVP and NA to overlap. The second case is assigned to the overlapping of NA and PVP as well as the interaction between the surface of silver nanoparticles and PVP through coordination bonding with the O and N atoms [47,50].

### 2.4. Thermal Analysis

**Figure 6:**
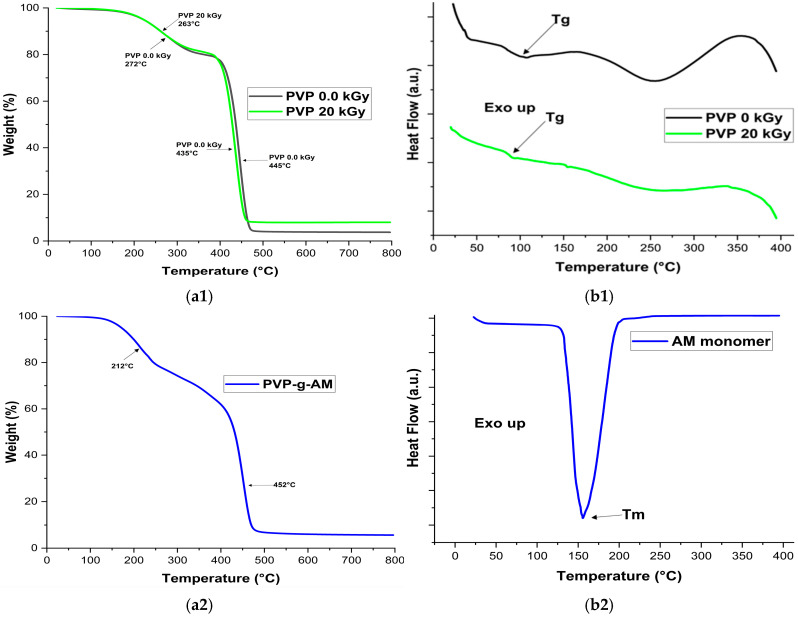

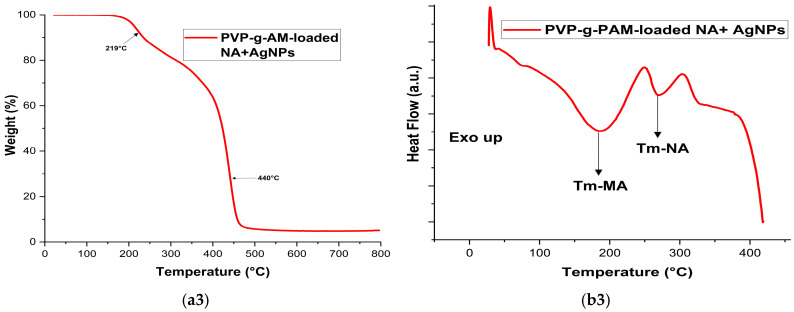
(**a1**) TGA analysis of PVP 0.0 kGy and PVP 20 kGy. (**a2**) TGA analysis of PVP-g-PAM. (**a3**) TGA analysis of PVP-g-PAM-loaded NA + AgNPs. (**b1**) DSC analysis of PVP 0.0 kGy and PVP 20 kGy. (**b2**) DSC analysis of AM monomer. (**b3**) DSC analysis of PVP-g-PAM-loaded NA + AgNPs.

Thermal analysis was carried out using thermogravimetric analysis (TGA) and differential scanning calorimetry (DSC). Figure 6(a1–a3) illustrates the results of the analysis by TGA. Initial weight losses of 10% were at about 262, 261, 199, and 234 °C for PVP 0.0 kGy, PVP 20 kGy, PVP-g-PAM, and PVP-g-PAM-loaded NA + AgNPs, respectively. Where from 25 to 100 °C is due to dehydration of the carboxylic acid group of MA [55], up this temperature is assigned to oligomers, low molecular weight, loss of moisture, and residual solvent into films [11]. PVP 0.0 kGy film presents two decomposition temperatures at 271 and 445 °C, obtaining a char yield (C, N_2_) of 6.6% at 800 °C. For PVP 20 kGy film, these temperatures of decomposition changed at 263 and 435 °C with a char yield of 8.14%. PVP-g-PAM showed lower decomposition temperatures at 212 and 452 °C, which could be ascribed to the presence of MA. Finally, PVP-g-PAM-loaded NA + AgNPs exhibit two decomposition temperatures observed at 219 and 440 °C with a char yield of 7.3% at 800 °C.

Figure 6(b1–b3) presents the DSC analysis. PVP 0.0 kGy and PVP 20 kGy films show water removal from 25 to 100 °C, while a glass transition temperature (Tg) is ~105 °C for PVP 0.0 kGy and 93 °C for PVP 20 kGy. In literature, Tg for PVP has been reported from 100 to 175 °C [16]; these differences in Tg are attributed to differences in molecular weight [16,56]. The peak up to ~350 °C indicates the beginning of polymer backbone degradation. MA monomer presents a melting point (Tm) at 155 °C; in literature, Tm for MA has been reported to be around 137 °C [57]. For PVP-g-PAM-loaded NA + AgNPs, two endothermic peaks are observed. The first point at ~190 °C is attributed to the Tm of MA, where the difference in Tm may be due to the change in molecular weight and formation of hydrogen bonds between PVP and MA [58]. The second point at ~269 °C is ascribed to the Tm of NA, which has been reported to be around ~250 °C [37,53].

### 2.5. Swelling Studies

**Figure 7:**
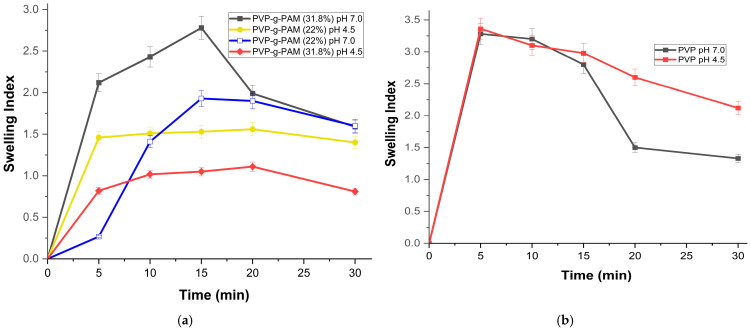

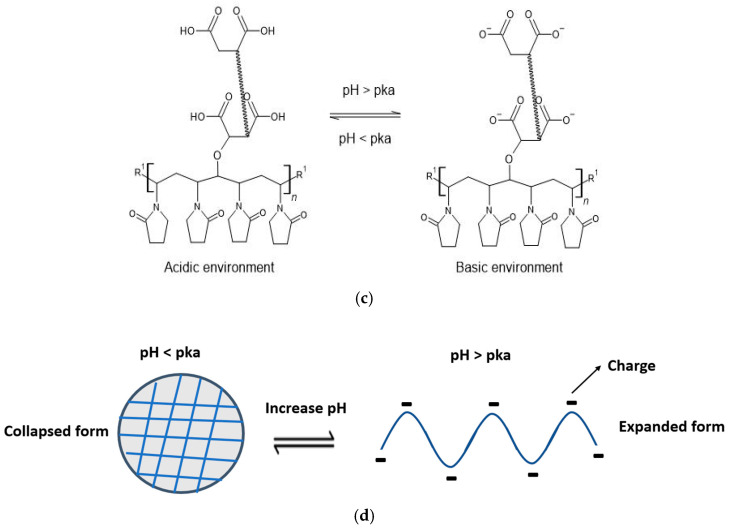
(**a**) Swelling index of PVP and PVP-g-PAM-loaded NA + AgNPs. (**b**) Swelling index of unmodified PVP. (**c**) Schematic representation of PVP-g-PAM in acidic and alkaline environments. (**d**) Example of pH response polymer.

Figure 7a presents the equilibrium swelling behavior of PVP-g-PAM-loaded NA + AgNPs as a function of pH at 4.5 and 7.0, considering the pKa values of MA (pKa1 = 1.83 and pKa2 = 6.07) reported by Jaiswal et al., 2005 [59]. As reported previously, the modified films with AM exhibited a pH-responsive behavior [28,30,32,33,34]. Due to the hydrophilic nature of the PVP, the films reached a limit of swelling at different times. After this limit, the films began to disperse in the aqueous medium, forming a gel and losing weight [60,61]. The film with 31.8% of AM grafting, followed by 22%, showed the greatest swelling when the pH was above pKa1 and pka2. This is because the film expands due to the complete ionization of both carboxylic acids of the AM [62]. Conversely, at a pH of 4.5, films with the same percentage of grafting exhibited reduced swelling. This is because only one carboxylic acid group (pKa1 = 1.83) is deprotonated, simultaneously causing the matrix to be in a semi-expanded and collapsed state. The latter is illustrated in Figure 7c,d. On the other hand, Figure 7b illustrates the swelling behavior of unmodified PVP at pH 7.0 and 4.5. It can be observed that PVP exhibits very similar behavior at both pH levels, as it does not have a pH-responsive structure.

### 2.6. Release In Vitro of NA

**Figure 8:**
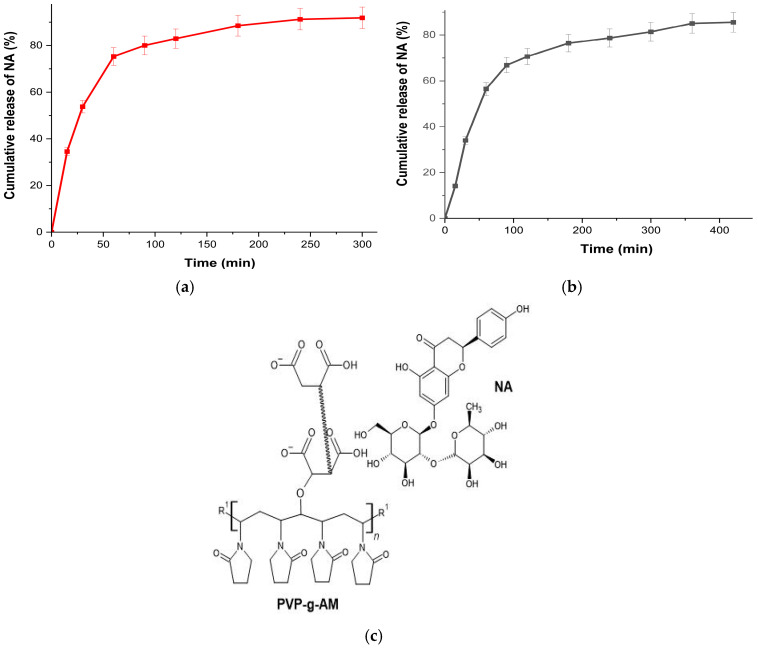
(**a**) Naringin release from PVP-g-PAM film at pH 7.0. (**b**) Naringin release from PVP-g-PAM film at pH 4.5. (**c**) Hydrogen bond interactions between AM and NA.

Figure 8a shows the results of NA release. At pH 7.0, a maximum release of 91% occurs at 240 min, while the release at pH 4.5 (Figure 8b) occurs more slowly, reaching a maximum of 85% at 360 min. This difference is attributed to the fact that at pH 4.5, only one of the two carboxylic acids is deprotonated (pka1 = 1.83), while the other remains protonated. Because of this, the protonated carboxylic acid forms hydrogen bond interactions as both donor and acceptor with NA [63] (Figure 8c), decreasing the release rate. Conversely, at pH 7.0, both carboxylic acids are deprotonated, resulting in only hydrogen bond interactions being donor from the NA side. The release profile of NA from films was performed by analyzing five kinetics models shown in Table 3, where Korsmeyer-Peppas was the one that shows a higher correlation coefficient, which implies that NA releases according to Fick’s Laws in which the diffusion flux occurs in the opposite direction to the concentration gradient [64].

### 2.7. Viscosimetry

**Figure 9:**
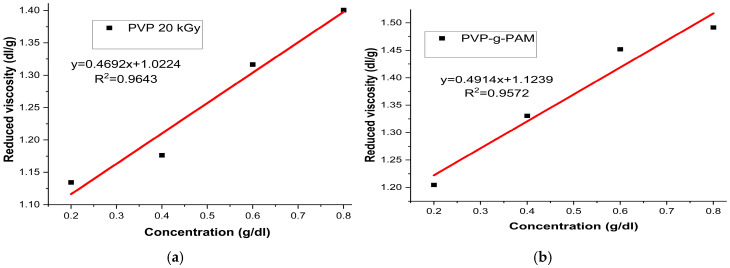
Graphs of reduced viscosity for PVP 20 kGy (**a**) and PVP-g-PAM (**b**).

Figure 9a,b present the graphs of reduced viscosity against the concentration of PVP irradiated at 20 kGy and PVP-g-PAM (25%), respectively. The PVP at 20 kGy graph showed a reduced viscosity as the concentration approached zero out of 1.0224 dL/g. In contrast, the PVP-g-PAM film exhibited a reduced viscosity of 1.1239 dL/g, suggesting an increase in molecular weight due to the grafting of AM.

### 2.8. Mechanical Properties

**Figure 10:**
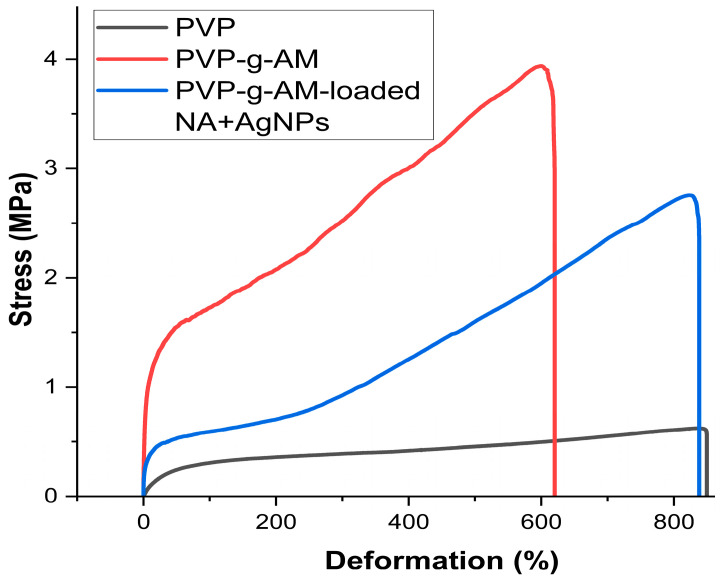
Graphs of stress deformation of PVP, PVP-g-PAM, and PVP-g-PAM-loaded NA + AgNPs films.

Figure 10 presents the graphs of mechanical properties for the different tested films, while the results are tabulated in Table 4. The PVP films exhibit a higher elongation percentage and, in turn, a lower Young’s modulus. This is attributed to the high hydrophilicity of PVP [65], which forms hydrogen bonds with water molecules [66], along with the presence of glycerol as a plasticizer that creates glycerol–PVP and glycerol–water interactions [67]. On the other hand, the PVP-g-PAM film shows lower malleability, resulting in reduced elongation due to the graft density, which leads to high entanglement molecular weights and extended backbone conformations caused by the steric repulsion of the side chains [68]. Finally, the PVP-g-PAM-loaded NA + AgNPs films exhibited greater elongation than PVP-g-PAM films. This is attributed to the presence of NA, which has 8 hydrogen donor bonds and 14 hydrogen acceptor bonds [63], resulting in a greater elongation.

### 2.9. Antimicrobial Studies

**Figure 11:**
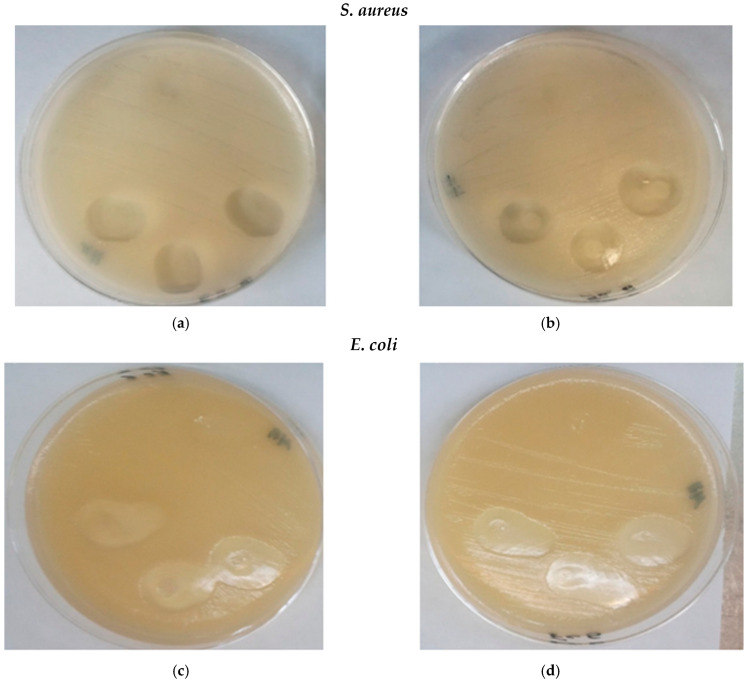
Results of antimicrobial test. (**a**) AgNPs—10 kGy, (**b**) AgNPs—30 kGy, (**c**) AgNPs—10 kGy, and (**d**) AgNPs—30 kGy.

The results of the antimicrobial test are presented in Table 5. PVP-g-PAM control films did not show any inhibition against the strains used; however, as expected, the addition of silver nanoparticles resulted in growth inhibition of both Gram-positive *S. aureus* and Gram-negative *E. coli*, as seen in Figure 11. There was no significant difference between silver nanoparticles synthesized at 10 and 30 kGy. The mechanism of action of AgNPs is unclear [69]; nonetheless, one mechanism proposed is the generation of ROS (Reactive Oxygen Species), which causes cellular stress and leads to bacterial death [70,71].

## 3. Conclusions

The graft copolymer PVP-g-PAM was performed using gamma ionizing radiation through the oxidative pre-irradiation method. In this way it was possible to obtain diverse films with different grafting percentages by adjusting the radiation dose and the exposition in a bath of water with the optimal conditions of 20 kGy and 80 min. Physicochemical characterizations such as DSC, TGA, and FTIR-ATR confirmed the modification of the films with AM, showing changes in the different films. The results from mechanical tests show that the films grafted with AM and loaded with NA and AgNPs exhibited an elongation greater than 700% and Young’s modulus of 11.29, indicating that the films possess good malleability for potential applications. Furthermore, the films demonstrated a pH-responsive behavior at various grafting percentages. The loading of silver nanoparticles adequately functioned as an antibacterial agent, inhibiting Gram-positive and Gram-negative strains. This together makes the modified films function as potential wound dressings, especially in burns, because there is no hemostasia process. Despite the promising results obtained, further studies are recommended to explore some key aspects in greater detail. Firstly, analyzing the surface topography using techniques such as atomic force microscopy (AFM) and scanning electron microscopy (SEM) will provide a better understanding of the physical and structural properties of the grafted films. Furthermore, evaluating the cytotoxicity of the materials through in vitro studies will be important to confirm their safety for use in medical applications. Finally, in vivo studies, particularly in animal models, must assess the material’s behavior in a real biological environment. This will provide critical information on its effectiveness and potential for clinical integration.

## 4. Materials and Methods

### 4.1. Materials

Polyvinylpyrrolidone (360 kDa), maleic acid (>99%), glycerol (99%), and naringin (>95%) were purchased from Sigma-Aldrich Co. (St. Louis, MO, USA). Absolute ethanol was acquired from Quimica Rique (Ecatepec de Morelos, Mexico). THF was obtained from J.T. Baker (Center Valley, PA, USA). AgNO_3_ (99%) was acquired from DEQ (Nuevo León, Mexico). All chemical compounds and solvents were used as received.

### 4.2. Formation of PVP Gels/Films

PVP gels were prepared by dissolving 1.2 g of PVP in a mixture of 42 mL ethanol, 0.3 g of glycerol as a plasticizer, and 16 mL of distilled water. Once a homogeneous gel was obtained, films were produced using the Casting method. The gel was poured into a silicone mold (46 cm^2^), covered with aluminum foil (previously perforated with small holes), and left to dry at room temperature. Once the films (thickness 0.3 mm ± 0.03) formed, they were cut into 1 × 5 cm pieces for further treatments.

### 4.3. Synthesis of PVP-g-PAM by Gamma Radiation

Films were dried under vacuum for over 5 h at 70 °C before recording initial weight (*Wo*). The films were placed into glass ampoules and exposed to a ^60^Co γ-source (Gammabeam 651 PT, MDSNordion, Ottawa, ON, Canada) in the presence of air at room temperature at a dose rate of 13.7 kGy h^−^¹, with doses ranging from 10 to 25 kGy. Subsequently, a saturated solution of 15% (*w*/*v*) of MA in THF was added into ampoules, which were then pumped with argon for 15 min and then sealed to generate an inert atmosphere. Then, the samples were heated in a water bath at 50 °C in reaction times ranging from 50 to 80 min. Afterward, films were extracted and soaked in THF for 30 min to remove residual monomer and homopolymer formed during the grafting process. After 24 h, films were under vacuum for 5 h at 70 °C before recording final weight (*Wf*). The grafting yield (*Yg*) was calculated as follows:

(1) $$Yg(\%)=\frac{\left(Wo-Wf\right)}{Wo}\times 100$$

where *Wf* = final weight of the grafted sample and *Wo* = initial weight of the pristine sample.

### 4.4. Preparation of AgNPs

AgNPs were prepared based on the method reported by Afify et al., 2017 [48]. A total of 0.1 g of PVP was dissolved in 20 mL of distilled water, and then 20 mL of freshly prepared AgNO_3_ solution (10 mM) was added to the PVP solution with continuous stirring. Afterward, the obtained solution was placed into test tubes and exposed to a ^60^Co γ-source (Gammabeam 651 PT, MDSNordion) at a dose rate of 13.7 kGy h^−1^ with doses of 10 and 30 kGy.

### 4.5. Load of NA and AgNPS

The load of NA was based on methodologies reported by Aggarwal et al., 2013, and Devi et al., 2003 [41,72,73]. Three PVP-g-PAM films and 5% naringin (relative to the weight of the 3 films) were dissolved in 15 mL of an ethanol/water (2:1) mixture, and then 75 µL of AgNPs were added with continuous stirring. Finally, the homogeneous solution obtained was poured into Teflon molds (20 cm^2^), covered with aluminum foil, and left to dry at room temperature.

### 4.6. In Vitro Release Studies

To prepare pH 4.5 and 7.0 buffer solutions, different quantities of boric acid, citric acid, and trisodium orthophosphate were weighed into beakers and transferred to a one-liter volumetric flask. The pH of the solutions obtained was then measured and adjusted, as appropriate, with acidic or basic buffer solution. Subsequently, 15 mg of each film was poured into glass bottles containing 25 mL of buffer solution at pH 4.5 and 7.0, respectively. The samples were then placed in a water bath at 37 °C and 60 rpm. To evaluate the progressive release of the drug, 2 mL aliquots were taken and filtered through a stainless steel mesh (sieve size approximately 33 µm) before being transferred to 3.5 mL quartz cuvettes (after measurement, the aliquot was returned to the release medium). Absorbance measurements were performed using the buffer solution as a reference, with a SPECORD^®^ 200 Plus UV-Vis spectrophotometer (Analytik Jena AG, Jena, Germany).

### 4.7. FTIR-ATR

Samples of PVP, MA, NA, PVP-g-PAM, PVP-g-PAM-loaded NA, and PVP-g-PAM-loaded NA + AgNPs were dried for 12 h at 60 °C. Afterward, samples were analyzed in a Perkin-Elmer Spectrum 100 Spectrophotometer with a diamond tip from Perkin Elmer Cetus Instruments, Norwalk, CT, USA, performing 16 scans for each using the ATR modulus.

### 4.8. TGA

Approximately 3–20 mg of each sample of PVP and PVP-g-PAM-loaded NA + AgNPs were dried for 24 h at 60 °C. Then, samples were placed on the platinum tray of the thermogravimetric analysis equipment TGA Q50 from TA Instruments, New Castle, DE, USA. Experiments were carried out in the temperature range from 25 to 800 °C under a nitrogen atmosphere, with a heating rate of 10 °C min^−1^.

### 4.9. DSC

Approximately 3–6 mg of each sample of PVP and PVP-g-PAM-loaded NA + AgNPs were dried for 24 h at 60 °C. Runs were recorded from 25 to 400 °C at a heating rate of 10 °C min^−1^, under a nitrogen atmosphere using a DSC 2010 calorimeter (TA Instruments, USA), starting at room temperature.

### 4.10. Swelling Measurement

The swelling index was calculated based on the method reported by Kumar et al., 2014 [20]. PVP-g-PAM-loaded NA + AgNPs films were cut into a surface area of 3 cm^2^, dried for 72 h at 60 °C, weighed, and transferred onto a stainless steel mesh (sieve size approximately 33 µm). Films were immersed in pH solutions of 4.5 and 7.0 at intervals (every 5 min); the stainless steel mesh was separated, excess water was removed carefully with paper, and the films were weighed. The swelling index was calculated by using the following formula:

(2) $$SI=\frac{\left(Wt-Wo\right)}{Wo}$$

where *SI* = swelling index, *Wt* = the weight of swelling film at time (*t*), *Wo* = weight of dried films.

### 4.11. Measurement Viscosimetry

Fifty milliliters of PVP and PVP-g-PAM solutions (concentrations ranging from 0.2 to 1 g/dL) were prepared. Measurements were conducted using an Oswalt viscometer from TA Instruments, New Castle, DE, USA, which was immersed in a water bath maintained at 25 °C.

### 4.12. Measurement Mechanical Properties

Films of 1 × 5 cm were stored at 55 ± 5% of relative humidity and at 22 °C ± 2 prior to the test. A tensile test was conducted using a Shimadzu Precision Universal/Tensile Tester from Long Beach, California, U.S.A with a constant elongation rate of 10 mm/min.

### 4.13. Antimicrobial Test

The antibacterial activity of films was evaluated against *S. aureus* and *E. coli* by the Kirby–Bauer method. The procedure involved preparing Hinton Müeller agar by adjusting the pH to 7.0; the medium was sterilized in an autoclave at 121 °C, 15 lb for 15 min. Then, the medium was cooled at 45 °C and was placed in Petri dishes for solidification. Afterwards, Petri dishes were placed in a 35 °C incubator for 24 h.

Two bacterial strains, *S. aureus* ATCC 25923 and *E. coli* ATCC 25922, were activated from deep freeze storage and cultured in their respective broths. Their bacterial concentration was adjusted to 0.5 MacFarland, equivalent to 1.5 × 10^8^ bacteria/mL. The bacterial concentration was determined using the surface extension technique. After changing the bacterial suspension to 0.5 MacFarland, six serial dilutions were made, and 100 μL from the last three dilutions (10^−4^, 10^−5^, 10^−6^) were plated on Hinton Müeller agar. The inoculum was spread with sterile glass beads, and the dishes were incubated at 35 °C for 24 h. After incubation, the colony count was used to calculate the bacterial concentrations, which were 4.7 × 10^7^ for *E. coli* and 5.1 × 10^7^ for *S. aureus*. A swab from the standardized bacterial tubes was used to inoculate the surface of the culture medium in Petri dishes, samples were placed in triplicate, and the dishes were incubated at 35 °C for 24 h. After incubation, inhibition halos were measured.

## Figures and Tables

**Table 1:** Grafting of MA onto PVP films. Varying dose.

Dose (kGy)	AM (% *w*/*v*)	Solvent	Grafting (%) ^(a)^	Temperature (°C)	Time (min)
10	15	THF	23.1 ± 3	50	50
15	15	THF	22.5 ± 3	50	50
20	15	THF	25.1 ± 2	50	50
25	15	THF	21.9 ± 2	50	50

^(a)^ Values are given as an average of 6 samples. (±Standard deviation).

**Table 2:** Grafting of MA onto PVP films. Varying time.

Dose (kGy)	AM (% *w*/*v*)	Solvent	Grafting (%) ^(b)^	Temperature (°C)	Time (min)
20	15	THF	25.1 ± 2	50	50
20	15	THF	31.1 ± 3	50	60
20	15	THF	31.8 ± 2	50	70
20	15	THF	17.6 ± 2.	50	80

^(b)^ Values are given as an average of 6 samples. (±Standard deviation).

**Table 3:** Kinetics models analyzed from PVP-g-PAM-loaded NA + AgNPs.

Model Kinetics	Correlation Coefficient (R^2^) at pH 4.5	Correlation Coefficient (R^2^) at pH 7.0
Korsmeyer-Peppas	0.91	0.86
Zero order	0.66	0.60
First order	0.85	0.85
Higuchi	0.87	0.84
Hixon-Crowell	0.66	0.60

**Table 4:** Mechanical properties of PVP, PVP-g-PAM, and PVP-g-PAM-loaded NA + AgNPs films.

Film	Tensile Strength (MPa) ^(a)^	Elongation at Break (%) ^(b)^	Young Modulus (MPa) ^(c)^	Thickness (mm) ^(d)^
PVP	0.71 ± 0.11	812 ± 25	0.825 ± 0.23	0.30 ± 0.03
PVP-g-PAM	3.86 ± 0.03	600 ± 9	20.92 ± 0.94	0.29 ± 0.01
PVP-g-PAM-loaded NA + AgNPs	3.0 ± 0.32	770 ± 54	11.29 ± 5.28	0.30 ± 0.02

^(a–d)^ Values are given as an average of 3 samples. (±Standard deviation). PVP-g-PAM and PVP-g-PAM-loaded NA + AgNPs films contained 31.8% grafting of MA.

**Table 5:** Antimicrobial results of PVP-g-PAM-loaded NA + AgNPs against *S. aureus* and *E. coli*.

	*S. aureus*	*E. coli*
AgNPs	Clear zone (mm)	Clear zone (mm)
10 kGy	15 ± 0.09	17.6 ± 1.1
30 kGy	16.8 ± 0.07	17.3 ± 0.45

Values are given as an average of 3 samples. (±Standard deviation). PVP-g-PAM-loaded NA + AgNPs contained 31.1% grafting of MA.

## Data Availability

The original contributions presented in this study are included in the article. Further inquiries can be directed to the corresponding authors.
